# A Survey on Entropy and Economic Behaviour

**DOI:** 10.3390/e22020157

**Published:** 2020-01-29

**Authors:** Ziv Hellman, Ron Peretz

**Affiliations:** Department of Economics, Bar-Ilan University, 5290002 Ramat Gan, Israel; Ron.Peretz@biu.ac.il

**Keywords:** bounded rationality, bounded memory, repeated games, entropy method

## Abstract

Entropy plays a significant role in the study of games and economic behaviour in several ways. A decision maker faced with an n-fold repetition of a decision-making problem needs to apply strategies that become increasingly complex as n increases. When several players are involved in selecting strategies in interactive games, bounds on the memories and cognitive capacities of the players can affect possible outcomes. A player who can recall only the last *k* periods of history is said to have bounded recall of capacity *k*. We present here a brief survey of results of games played by players with different bounded recall capacities, in particular those indicating surprisingly strong relations between memory and entropy in the study of the min-max values of repeated games with bounded recall. In addition, we consider uses of entropy in measuring the value of information of noisy signal structures, also known as experiments. These are represented by stochastic matrices, with the rows representing states of the world and the columns possible signals. The classic ordering of experiments, due to David Blackwell and based on decision-making criteria, is a partial ordering, which has led to attempts to extend this ordering to a total ordering. If a decision maker has a prior distribution over the states, receipt of a signal yields a posterior. The difference between the entropy of a prior and the expected entropy of the set of possible posteriors has been proposed as a natural extension of the Blackwell ordering. We survey this alongside the theory of rational inattention, which posits that, since individuals have limited attention, they do not always follow every single piece of economic news in planning their economic behaviour. By modelling attention limits as finite channel capacity in the sense of Shannon, economists have developed a theory that explains a range of observed economic behavioural phenomena well.

## 1. Introduction: The Exam Example

We recently taught a course on the usage of information theory in game theory. It did not take long for the students to get to the main point; on the first day of classes they asked us:

what do we really need to know in order to receive a first class grade?

Stunned by their directness, we tried to elude the matter by giving them all sorts of evasive replies but still they insisted, so eventually we gave up and bluntly told them the truth:

There will be a multiple-choice examination comprised of thirty questions, with a choice of four possible answers (only one of which will be correct) for each question. That is what you *really* need to know!

…oh, and here is the list of correct answers:

a b d c b b c a b d a b d b a d b a b d b d b c a b d b a b.

After a pause (and after erasing the list of correct answers from the board), we continued

Okay, now that we’re on the same page, can we start? Well, then… please close all your notebooks and electronic devices. The exam begins now!

Obviously, most of our students would not have done very well had we really given them the exam at that point, even though we had revealed *all* of the information they needed in order to receive perfect marks! That is probably because they were not ready, and therefore could not process the information they were given sufficiently quickly. Taking pity on them, we decided to give them a second chance. This time, we warned them in advance what was going to happen, namely, that we were about to repeat the same scenario again. We would announce the correct answers and then they would take the exam, but they would be allowed enough time to prepare strategies before the correct answers were revealed.

What were the outcomes of the experiment? Different students succeeded differently in the ‘exam’, perhaps in part because they differed in their memory capacities and in part because they applied different strategies for recalling the information regarding the correct answers.

Asking what can or cannot be done given one’s cognitive abilities, or merely how to model this situation, led us to the true lesson of Week 1 of the course. The whole purpose of the experiment was to introduce a model of decision making that would be used throughout the course. That model, which is described in Section 2.1 below, is simple enough to be solved analytically, yet also rich enough to demonstrate the use of information theory in decision theory (which can also be termed ‘one-player game theory’) as well as to facilitate the analysis of more complicated models that were throughout the course. These models will be outlined in Section 2 next.

## 2. Bounded Rationality and Coding Theory

### 2.1. A Simple Decision Making Model

We start with some standard definitions used in decision theory. A *decision making problem* consists of three components:

(i) a finite set *S* of (all possible) states of the world,
(ii) a finite set *A* of (all possible) actions,
(iii) a utility function u:S×A→R.

A single question in the above exam example can be modelled as follows: S=A={a,b,c,d} is the set of all possible answers. The state is the correct answer and the action is the student’s answer. The utility function is

$$u\left(s,a\right)=\left\{\begin{array}{cc} 1 & \mathrm{if}\;s=a,\\ 0 & \mathrm{if}\;s\neq a. \end{array}\right.$$

To model an exam of thirty questions one needs a notion of a repeated problem. The utility function of the *n*-fold repetition of the problem $u_{n}:S^{n}\times A^{n}\to R$ is defined as

$$u_{n}\left(\vec{s},\vec{a}\right)=\frac{1}{n}\sum_{i=1}^{n}u\left(s_{i},a_{i}\right),$$

where $\vec{s}=\left(s_{1},\ldots ,s_{n}\right)$ and $\vec{a}=\left(a_{1},\ldots ,a_{n}\right)$.

A (pure) *strategy* in the *n*-fold repetition is a mapping $\sigma_{n}:S^{n}\to A^{n}$. The *worth* of a strategy is the utility it guarantees (in the worst case)

$$v\left(\sigma_{n}\right)=\min_{\vec{s}\in S^{n}}u_{n}\left(\vec{s},\sigma_{n}\left(\vec{s}\right)\right).$$

We would like to capture the idea of cognitive capacity in the framework of our model. A rather simple (one may say “simplistic”) yet useful way to do so is to consider the cardinality of the image of strategies. The *(image-size) complexity* of a strategy is defined as

$$\mathrm{comp}\left(\sigma_{n}\right)=\left|\sigma \left(S^{n}\right)\right|.$$

The *(k,n)-value* of a decision making problem *u* is defined as

$$Val\left(u,k,n\right)=\max\{v\left(\sigma_{n}\right):\mathrm{comp}\left(\sigma_{n}\right)\leq k\}.$$

In game-theoretic terms (Here, we refer to *u* as a two-person zero-sum game between players who choose actions in *A* and *S* respectively. A formal definition of a two-person zero-sum game is given in Section 2.4), Val(u,k,n) is the pure max-min value of the *n*-fold repetition of *u* restricted to strategies whose image-size is at most *k*.

Considering the exam example, it is not hard to see that for k≤3, Val(u,k,n)=0. Indeed, for any three sequences of actions, there is a sequence that differs from each one of the three in any position. For k=4, the decision make can ensure the utility of $\frac{1}{4}$ by repeating a single action throughout, the action that matches the most frequent state. How well can the decision maker do with k=5, or k=100, or $k=n^{2}$? Theorem 1 below estimates Val(u,k,n) as a function of the ratio $\frac{\log k}{n}$. In particular, *k* polynomial in *n* is not much better than k=|S|. In order to do better than that, *k* needs to be exponential in *n*.

Before we provide the general estimate, let us first analyse the exam example through an information-theoretic lens. Given *n* and *k* an optimal strategy is a choice of *k* points in the Hamming space $A^{n}$, such that the balls of (normalised) radius Val(u,n,k) around these points cover the entire space. Coding theory tells us that we can choose such balls to be “almost” pairwise disjoint, and so estimating Val(u,n,k) boils down to estimating the size of a Hamming ball of a give radius.

Our estimate of the image-size value is analogous to Shannon’s channel capacity. In order to describe the estimate, consider the following two-player game between nature and the decision maker (DM) parameterised by a non-negative real number *h*. The game is played in two stages. In Stage 1 nature chooses a probability distribution over the states of the world p(s). In Stage 2, DM chooses, for every possible state *s*, a conditional distribution p(a|s) over the set of actions. The moves of nature and DM induce a joint distribution p=p(s,a)=p(s)p(a|s) over S×A. DM’s choice is subject to the restriction that the mutual information of the state and the action (with respect to *p*) is bounded by *h*. The joint distribution over states and actions induces a distribution over utilities. The goal of nature is to minimise the expected utility and the goal of DM is to maximise it. The value of this game (i.e., the unique equilibrium payoff) is denoted v(u,h). Formally,

$$v\left(u,h\right)=\min_{p(s)}\max_{\begin{array}{c} p(a|s):\\ I_{p}\left(s;a\right)\leq h \end{array}}{\mathrm{error}}_{p}u\left(s,a\right),$$

where p(s) ranges in Δ(S), p(a|s) ranges in ${\left(\Delta \left(A\right)\right)}^{S}$, p=p(s,a)=p(s)p(a|s) and Δ(X) denotes the set of all probability measures on the finite set *X*.

The following theorem is analogous to Shannon’s noisy-channel coding theorem.

**Theorem** **1.** *DM cannot do better than $v(u,\frac{\log k}{n})$, but can do better than $v(u,\frac{\log k}{n})-\varepsilon$, for any ε>0 and k large enough. Namely,*

$$0\leq v\left(u,\frac{\log k}{n}\right)-Val\left(u,k,n\right)\to 0,$$

*as k→∞ (uniformly in n).*

The proof of Theorem 1 is outlined in Section 2.2 below.

*Randomisation helps?*

**Remark** **1.** *What happens if DM is allowed to randomise? In the exam example with k=1, by randomising uniformly between the four pure constant-action strategies, a mixed strategy guarantees an expected utility of $\frac{1}{4}$, whereas any pure strategy guarantees 0 utility (Val(u,k,n)=0, for k≤3). So, for some values of k, randomisation helps. Nevertheless, denoting the randomised (k,n)-value by $Val^{\prime }\left(u,k,n\right)$, one has that $Val\left(u,k,n\right)\leq Val^{\prime }\left(u,k,n\right)\leq v\left(u,\frac{\log k}{n}\right)$, for any u, k and n; therefore for large values of k and n, randomisation makes only a negligible difference.*

### 2.2. Neyman–Okada Lemma

In a sequence of papers, Neyman and Okada [1,2,3] developed a methodology for analysing repeated games with bounded memory. A key idea of theirs is captured in a simple potent lemma. Even though the proof of Theorem 1 does not require the full generality of the Neyman-Okada Lemma, we take this proof as an opportunity to demonstrate a utilisation of the lemma.

**Lemma** **1** (Neyman–Okada)**.** *Let $x_{1},\ldots ,x_{m},y_{1},\ldots ,y_{m}$ be finite random variables and let $y_{0}$ be a random variable such that each $y_{i}$ is a function of $y_{0},x_{1},\ldots ,x_{i-1}$. Suppose that t is a random variable that distributes uniformly in $\left\{1,\ldots ,m\right\}$ independently of $y_{0},x_{1},\ldots ,x_{m},y_{1},\ldots ,y_{m}$. Then,*

$$I\left(x_{t};y_{t}\right)\leq H\left(x_{t}\right)-\frac{1}{m}H\left(x_{1},\ldots ,x_{m}\right)+\frac{1}{m}I\left(y_{0};x_{1},\ldots ,x_{m}\right).$$

**Proof.**  $$\begin{array}{c} I\left(x_{t};y_{t}\right)-H\left(x_{t}\right)=-H\left(x_{t}|y_{t}\right)\leq -H\left(x_{t}|y_{t},t\right)=H\left(x_{t}\right)-\frac{1}{m}\sum_{i=1}^{m}H\left(x_{i}|y_{i}\right)\\ \leq -\frac{1}{m}\sum_{i=1}^{m}H\left(x_{i}|y_{i},x_{i-1},\ldots ,x_{1},y_{0}\right)=-\frac{1}{m}\sum_{i=1}^{m}H\left(x_{i}|x_{i-1},\ldots ,x_{1},y_{0}\right)\\ =-\frac{1}{m}H\left(x_{1},\ldots ,x_{m}|y_{0}\right)=-\frac{1}{m}H\left(x_{1},\ldots ,x_{m}\right)+\frac{1}{m}I\left(y_{0};x_{1},\ldots ,x_{m}\right). \end{array}$$

 □

The Neyman–Okada Lemma provides an upper bound for the amount of inter-dependence between $x_{t}$ and $y_{t}$ (measured by mutual information) as a sum of two terms: $H\left(x_{t}\right)-\frac{1}{m}H\left(x_{1},\ldots ,x_{m}\right)$ and $\frac{1}{m}I\left(y_{0};x_{1},\ldots ,x_{m}\right)$. The first term depends only on $\vec{x}=\left(x_{1},\cdots ,x_{m}\right)$; it measures how close $\vec{x}$ is from being a sequence of i.i.d. random variables. The second term measures the amount of information that $y_{0}$ has on $\vec{x}$. The second term can be bounded from above by the entropy of $y_{0}$, which is a quantity that depends only on $y_{0}$. Thus, the lemma bounds the interdependence between $x_{t}$ and $y_{t}$ by the sum of two terms, one of which depends only on $\vec{x}$ and the other only on $y_{0}$. Of special interest is the case where $x_{1},\ldots ,x_{m}$ are i.i.d. and $y_{0}$ has small support. In this case we have

(1) $$I\left(x_{t};y_{t}\right)\leq \frac{1}{m}\log(|\mathrm{supp}\left(y_{0}\right)\left|\right).$$

**Proof** **of** **Theorem** **1.** In one direction, we prove the stronger statement of Remark 1, that is, we show that $Val^{\prime }\left(u,k,n\right)\leq v\left(u,\frac{\log k}{n}\right)$, for any *u*, *k* and *n*. Let p∈Δ(S) be a minimiser of the expression that defines $v(u,\frac{\log k}{n})$. Let $p^{n}\in \Delta \left(S^{n}\right)$ be the *n*-fold product of *p* with itself. Let $\Sigma \left(k,n\right)=\{\sigma_{n}:\sigma_{n}:S^{n}\to A^{n}\;\mathrm{and}\;|\sigma_{n}\left(S^{n}\right)|\leq k\}$. Since, by Von Neumann’s min-max theorem,

$$\begin{array}{cc} Val^{\prime }\left(u,k,n\right) & =\max_{\sigma_{n}^{\prime }\in \Delta \left(\Sigma (k,n)\right)}\min_{\vec{s}\in S^{n}}\underset{\sigma_{n}∼\sigma_{n}^{\prime }}{\mathrm{error}}u_{n}\left(\vec{s},\sigma_{n}\left(\vec{s}\right)\right)\\ \; & =\min_{s^{\prime }\in \Delta \left(S^{n}\right)}\max_{\sigma_{n}\in \Sigma \left(k,n\right)}\underset{\vec{s}∼s^{\prime }}{\mathrm{error}}u_{n}\left(\vec{s},\sigma_{n}\left(\vec{s}\right)\right), \end{array}$$

it is sufficient to show that ${\mathrm{error}}_{\vec{s}∼p^{n}}u_{n}\left(\vec{s},\sigma_{n}\left(\vec{s}\right)\right)\leq v\left(u,\frac{\log k}{n}\right)$, for every $\sigma_{n}\in \Sigma \left(k,n\right)$.

We apply the Neyman–Okada Lemma with $\left(x_{1},\ldots ,x_{n}\right)∼p^{n}$, $y_{0}=\left(y_{1},\ldots ,y_{n}\right)=\sigma_{n}\left(x_{n}\right)$, and *t* uniformly distributed in {1,…,n} independently of the other random variables. By Inequality 1 and, since $|\mathrm{supp}(y_{0})|\leq k$, we have

$$I\left(x_{t};y_{t}\right)\leq \frac{\log k}{n}.$$

Let q(s,a)∈Δ(S×A) be the joint distribution of $\left(x_{t},y_{t}\right)$. The proof is concluded by

$$\begin{array}{cc} {\mathrm{error}}_{\vec{s}∼p^{n}}u_{n}\left(\vec{s},\sigma_{n}\left(\vec{s}\right)\right) & =\mathrm{error}u\left(x_{t},y_{t}\right)={\mathrm{error}}_{q}u\left(s,a\right)\\ \; & \leq \max_{\begin{array}{c} q(s,a):\\ q(s)=p,\\ I_{q}\left(s;a\right)\leq h \end{array}}{\mathrm{error}}_{q}u\left(s,a\right)=\min_{p^{\prime }}\max_{\begin{array}{c} q(s,a):\\ q\left(s\right)=p^{\prime },\\ I_{q}\left(s;a\right)\leq h \end{array}}{\mathrm{error}}_{q}u\left(s,a\right)\\ & =v\left(u,\frac{\log k}{n}\right). \end{array}$$

 □

### 2.3. Refinements of the Simple Model

In this section we present more restrictive (and realistic) measures of complexity than the image size. Surprisingly, the conclusion of Theorem 1 will hold even with respect to the more restrictive models.

Image size is the number of different reactions a person (strategy) may have to different stimuli. Having different reactions to different stimuli means that the person necessarily distinguishes between the stimuli, that is, image size measures the number of different classes of stimuli that can be distinguished *de facto*. In reality, however, some pairs (or sets) of inputs are harder to distinguish than others. Alas, the image-size complexity measure is too coarse to make this distinction.

Let us try to capture the idea of bounded cognitive capacity more realistically. Image-size complexity assumes that the input is given in one piece. In reality, we read the input bit-by-bit (say, left to right) and process it sequentially. We need to maintain a cognitive image of the input as we process it. By the time we finish reading the input, we reach a certain cognitive image in our mind from which we form a reaction.

The formal model that corresponds to the above description is that of a finite automaton (a.k.a. Kripke structure). A finite automaton consists of a finite set of states, an initial state (one of the states) and a transition function that maps each pair of states and input letter to a state. One way to turn a finite automaton into a strategy, is to specify a mapping from the states to outputs (sequences of actions). The *automaton-size complexity* of a strategy is the minimal number of states needed in order to implement it through a finite automaton.

Clearly, the automaton-size complexity of any strategy is not smaller than its image-size complexity. Nevertheless, the corresponding value notions are asymptotically equal. The logic behind this is that an asymptotically optimal strategy of image size *k* can be simulated by an automaton of roughly the same size as follows: the input is read in small chunks; each input chunk is temporarily memorised; then, an optimal reaction to that chunk is figured out (with respect to the ratio (logk)/n); the temporary memory is discarded while retaining only the concise reaction; then, the next chunk is processed in the same fashion and so on. At the end, the output of the automaton is the concatenation of all the reactions. Choosing the lengths of chunks much larger than 1 and much smaller than *n* ensures that the value is asymptotically guaranteed, and the number of additional states required (beyond *k*) is negligible in the exponential scale.

One may argue that this proposed model of automaton complexity is still not realistic, since assuming that any state can be associated with an arbitrary output sequence under-counts the true cognitive capacity required to implement the strategy. To answer such a critique, one may modify the model slightly as follows. Suppose each state of the automaton is associated with only one output action (rather than a sequence of *n* actions). As the automaton runs over the input, it generates an output according to states visited. The input is fed into the automaton twice in tandem. At each round a score (in utility units) is obtained as the function *u* of the input and output of that round. The final normalised utility is the average score in rounds n+1 through 2n. In particular, the output in the first *n* stages does not count towards the final score.

The respective value of the *modified automaton model* is still asymptotically equal to the image-size value. To see that, one can count the number of addition states needed in order to translate an automaton that outputs an entire sequence of actions into one that outputs one action at a time. It is sufficient to show that the size of the latter automaton is not bigger than $n^{2}$ times the size of the former, which is a negligible difference on the exponential scale. Such a translation can be done as follows. In the first phase, rounds 1 to *n*, each state is coupled with a clock that keeps track of the calendar time in order to detect the transition to the second phase, rounds n+1 to *n*. In the second phase, the automaton runs obliviously (i.e., ignoring the input) while outputting the sequence prescribed by the simulated automaton. Each one of the phases requires at most *n* states per state of the original automaton; hence the coupling of all these states multiplies the automaton size by $n^{2}$ at most.

### 2.4. Playing with Finite Automata

In a survey paper on repeated games, Aumann [4] introduced finite automata as a model of bounded rationality. We will present a special case of Aumann’s model, for the case of two-person zero-sum repeated games.

We begin with a few standard game-theoretic definitions. A two-person zero-sum game $G=\langle A=A_{1}\times A_{2},u:A\to R\rangle$ consists of the following components:

- a set of two players {1,2},
- finite sets of actions $A_{1}$ and $A_{2}$ of Players 1 and 2 respectively,
- a payoff function $u:A=A_{1}\times A_{2}\to R$ (that 2 pays 1).

An action profile is an element of $A=A_{1}\times A_{2}$. A history is a finite sequence of action profiles, that is, an element of $A^{<\infty }={⋃}_{t=0}^{\infty }A^{t}$. A (pure reduced) strategy for player *i* (i=1,2) is a function $\sigma^{i}:A_{-i}^{<\infty }\to A_{i}$. (This is a tolerable deviation from the standard definition of a strategy, which would be a function from $A^{<\infty }$ to $A_{i}$. The standard game-theoretic notation “−i” refers to the player(s) other than *i*, that is, 2−i.) The set of all strategies for player *i* is denoted $\Sigma^{i}$. A strategy profile is an element of $\Sigma^{1}\times \Sigma^{2}$. A strategy profile $\left(\sigma^{1},\sigma^{2}\right)$ induces a play $\vec{a}\in A^{\infty }$ defined recursively by $a_{1}^{i}=\sigma^{i}\left(\emptyset \right)$ and $a_{t+1}^{i}=\sigma^{i}\left(a_{1},\ldots ,a_{t}\right)$.

A strategy $\sigma^{i}$ and a history $h=\left(h_{1},\ldots ,h_{t}\right)\in A^{t}$ induce a continuation strategy $\sigma_{|h}^{i}\in \Sigma^{i}$, defined by

$$\sigma_{|h}^{i}\left(a_{1}^{-i},\ldots ,a_{k}^{-i}\right)=\sigma^{-i}\left(h_{1}^{-i},\ldots ,h_{t}^{-i},a_{1}^{-i},\ldots ,a_{k}^{-i}\right).$$

Note that the definition of $\sigma_{|h}^{i}$ ignores the actions of player *i* along the history *h*. In particular, *h* need not be consistent with $\sigma^{i}$.

A strategy $\sigma^{i}$ is said to have finite memory if it only has finitely many continuations, and the cardinality of its set of continuations is called the *memory size* complexity of $\sigma^{i}$, that is, the cardinality of the set $\left\{\sigma_{|h}^{i}:h\in A^{<\infty }\right\}$. The set of strategies of player *i* whose memory size is at most *k* is denoted $\Sigma^{i}\left(k\right)$.

Let *G* be a finite two-person zero-sum game *G*, $k^{1},k^{2}\in N$ and T∈N∪{∞}. The *T*-fold repeated version of *G* where each player *i* is restricted to strategies with $k^{i}$ memory size is denoted $G^{T}\left(k^{1},k^{2}\right)$, that is, $G^{T}\left(k^{1},k^{2}\right)$ is a finite two-person zero-sum game with action sets $\Sigma^{i}\left(k^{i}\right)$, i=1,2 and a payoff function $u_{T}:\Sigma^{1}\left(k^{1}\right)\times \Sigma^{2}\left(k^{2}\right)\to R$ defined as follows: for strategies $\sigma^{i}\in \Sigma^{i}\left(k^{i}\right)$, i=1,2, that induces a play $a_{1},a_{2},\ldots$,

$$\begin{array}{cccc} u_{T}\left(\sigma^{1},\sigma^{2}\right) & =\frac{1}{T}\sum_{t=1}^{T}u\left(a_{t}\right), & \; & \left(T<\infty \right)\\ u_{\infty }\left(\sigma^{1},\sigma^{2}\right) & =\lim_{T\to \infty }u_{T}\left(\sigma^{1},\sigma^{2}\right). & \; & \end{array}$$

The above limit exists since the play must enter a loop after at most $k^{1}\times k^{2}$ periods.

**Remark** **2.** *Memory- and automaton-size complexities are often used inter-changeably in the literature. The reason for this is that a finite-memory strategy for player i can be implemented through a finite automaton whose input and output alphabets are $A_{-i}$ and $A_{i}$, respectively. Furthermore, the memory-size complexity of a strategy is the size of the smallest automaton that implements it (with respect to the modified automaton model). See Section 3 in Reference [5].*

The (mixed strategy) max-min value of the game $G^{T}\left(k^{1},k^{2}\right)$ has been a natural subject of investigation since the introduction of the model in the early 1980s. Ben-Porath [6] showed that as long as $\lim_{n\to \infty }\frac{k^{i}\left(n\right)}{k^{-i}\left(n\right)}=0$, i=1,2, $\lim_{n\to \infty }Val^{\prime }G^{\infty }\left(k^{1}\left(n\right),k^{2}\left(n\right)\right)=Val^{\prime }G$. Neyman [5] then showed that $\lim_{k\to \infty }Val^{\prime }G^{\infty }\left(k,\exp\left(Ck\right)\right)=ValG$, for sufficiently large *C*. In general, characterising the (asymptotic) value of $G^{T}\left(k^{1},k^{2}\right)$ remains an open problem. Interesting particular cases include the following theorems.

**Problem** **1.** *It is not known what the values of $G^{\infty }\left(k,\exp\left(ck\right)\right)$ and $G^{ck\log k}\left(k,\infty \right)$ are, for an arbitrary (small) constant c>0.*

Neyman [3] took a step towards a solution of Problem 1 that generalises Ben-Porath’s result. An oblivious strategy is a strategy which ignores the actions of the other player, that is, it plays a certain sequence of actions regardless of what the other player is doing. Let $G^{T}\left(k^{1},k_{obl}^{2}\right)$ be the game derived from $G^{T}\left(k^{1},k^{2}\right)$ by further restricting Player 2 to oblivious strategies. Namely, Player 2 is restricted to playing sequences of actions that enter a loop of length $\ell \leq k^{2}$ after $\ell_{0}\leq k^{2}-\ell$ periods. That is, Player 2 plays sequences of the form abbbb…, where $a\in A_{2}^{\ell_{0}}$ and $b\in A_{2}^{\ell }$, $\ell \in \{1,\ldots ,k^{2}\}$, $\ell_{0}\in \left\{0,\ldots ,k^{2}-\ell \right\}$.

**Theorem** **2** (Neyman 2008 [3])**.** *For every stage game G=〈A,u〉, $k^{1},k^{2}\in N$ and T∈N∪{∞},*

$$Val^{\prime }G^{T}\left(k^{1},k_{obl}^{2}\right)\leq v\left(u;\frac{\log k^{1}}{k^{2}}\right),$$

*and*

$$ValG^{\infty }\left(k^{1},k_{obl}^{2}\right)\geq v\left(u,\frac{\log k^{1}}{k^{2}}\right)-\varepsilon ,$$

*for every ε>0 as long as $k^{1}$ is large enough.*

**Proof**  **outlines.** The first part follows from Neyman–Okada’s lemma. Take $p\in \Delta (A_{2})$ that attains the maximum in the definition of $v(u,\frac{\log k^{1}}{k^{2}})$. Player 2 plays a random $k^{2}$-periodic sequence ${\left(a_{t}^{1}\right)}_{t=1}^{\infty }$ such that any $k^{2}$ consecutive elements are independent *p* distributed. Let $\sigma^{1}\in \Sigma^{1}\left(k^{1}\right)$ be Player 1’s (best) response. Divide the duration of the game into epochs of length $k^{2}$. If *T* is finite and not divisible by $k^{2}$ then the first epoch will be shorter than $k^{2}$. Clearly, an expected payoff of at most $v(u,\frac{\log k^{1}}{k^{2}})$ is attained during the first epoch. Let us look at any other epoch which is of length $k^{2}$. Let *h* be the history of play before the beginning of the epoch and $a_{|h|+1},\ldots ,a_{\left|h\right|+k^{2}}$ the play during the epoch. Apply Neyman–Okada’s lemma with $x_{i}=a_{|h|+i}^{2}$, $y_{0}=\sigma_{|h}^{1}$ and $y_{i}=a_{|h|+i}^{1}$, $i=1,\ldots ,k^{2}$. The proof follows from Inequality (1), since $\sigma^{1}$ has at most $k^{1}$ different continuations.

The second part of the theorem is similar to the analysis of automaton-size complexity in Section 2.3, where Player 1 plays the role of the decision maker and Player 2 is Nature. The only difference is that here, the exact length of the period is not known; it can vary strategically between 1 and $k^{2}$. It turns out that the exact period length can be learnt using a small number of states (sub-exponential in $k^{2}$) which concludes the proof. □

The second part of Theorem 2 refers to the infinitely repeated game, that is, T=∞. One might wonder whether the same statement extends to finite values of *T* and, if so, to what extent. It turns out that the direct answer to this question is that $T=\omega (k^{2})$ is a sufficient and necessary condition. Generally, *T* cannot be $O(k^{2})$, since Player 2 can always play an optimal strategy during the first $k^{2}$ stages of the game.

A more interesting question to ask is how long it takes until Player 1 starts to receive the guaranteed payoff $v\left(u,\frac{\log k^{1}}{k^{2}}\right)-\varepsilon$. That is, consider a payoff function that discounts the first *T* periods, and takes into account the payoffs in any time interval T+1 through T+k, for $k\geq k^{2}$. How large need *T* be to secure the guaranteed payoff? Peretz [7] showed that $T\geq 5k^{2}$ is sufficient.

**Remark** **3.** *Learning the period length of a stream of input bits using finite memory is an interesting problem in its own right. Formally, the problem can be stated as follows—the input is an infinite periodic steam of bits (or letters in a given finite alphabet) with period length at most n. The output is the period length. Reference [8] found a probabilistic algorithm that runs in TIME 2n and SPACE $O({\left(\log n\right)}^{2})$ (equivalently, automaton-size $n^{O(\log n)}$). A deterministic algorithm has a TIME×SPACE trade-off of $\Omega (n^{2})$ ([7]). Thus, finding the period length in sub-linear SPACE (sub-exponential automaton-size) takes super linear TIME. However, in order to device the automaton for Player 1 in the proof of Theorem 2, it is sufficient to find a large number that divides the period length, and the latter problem is solvable in TIME 2n and SPACE $O(\sqrt{n\log n})$, as shown in Reference [7].*

## 3. Bounded Recall and High Entropy

In addition to the model of finite automata described in Section 2.4, Aumann [4] proposed another model of bounded memory called *bounded recall*. The model we describe in this note is slightly different from Aumann’s original model. Aumann defined bounded recall strategies as strategies that do not observe one’s own actions and rather only look at the other players’ actions. We refer to the more commonly studied definition that allows bounded recall to monitor all of the players.

The idea is simple. A finite game is played repeatedly. A player with capacity *k* recalls only the last *k* periods of history. Different players may have different recall capacities. The result is again a finite game, but much larger than the one-stage game. The number of *k*-recall strategies grows double exponentially in *k*. Interesting game-theoretic quantities such as equilibrium payoffs and pure/mixed max-min/min-max levels are challenging to calculate due to the immense number of strategies.

Although our understanding of bounded recall games is far from being complete, it is better than our understanding of bounded automaton size games. Here, we do have a few estimates for the value of the game. As the research progresses, it is becoming ever clearer that memory and entropy are related.

We turn now to describe the model of bounded recall starting with two-person zero-sum games. Let $G=\langle A=A_{1}\times A_{2},u:A\to R\rangle$ be a finite (one-stage) two-person zero-sum game. A *k*-recall strategy for player *i* is a mapping $\sigma^{i}:A^{<\infty }\to A_{i}$ that satisfies

$$\sigma^{i}\left(a_{1},\ldots ,a_{t}\right)=\sigma^{i}\left(a_{t-k+1},\ldots ,a_{t}\right),$$

for every t≥k and $a_{1},\ldots ,a_{t}\in A$.

The set of *k*-recall strategies for player *i* is denoted $\Sigma^{i}\left[k\right]$. The *T*-fold repetition of *G* where each player *i* is restricted to $k^{i}$-recall strategies is denoted $G^{T}\left[k^{1},k^{2}\right]$. Further restricting Player 1 to oblivious strategies yields the game $G^{T}\left[k_{obl}^{1},k^{2}\right]$.

**Remark** **4.** *Bounded recall can be viewed as a special case of bounded memory. Strictly speaking, their definitions need to be slightly modified in order to make such a comparison. The domain of a (k-recall) strategy is defined as $A^{<\infty }$ rather than $A_{-i}^{<\infty }$ as in the definition of a reduced strategy that was used for the definition of memory-size in Section 2.4. Other than that difference, a k-recall strategy can be viewed as a strategy with memory size ${\left|A\right|}^{k}$, since it can be implemented through a finite automaton whose state is the history of the last k action profiles. The other direction does not hold, that is, a bounded memory strategy need not have bounded recall capacity. In particular, the “state” of a bounded recall strategy is observable by the other player, whereas the state of a bounded memory strategy is not. Even though bounded recall can be viewed as a special case of bounded memory, results in one model do not translate automatically to results in the other model, since restricting *both* players to subsets of their strategies may result in changing the value of the game in either direction, up or down.*

The mixed value of $G^{T}\left[k^{1},k^{2}\right]$ is estimated as a function of $\frac{\log k^{2}}{k^{1}}$ (assuming without loss of generality that $k^{1}\leq k^{2}$). The function ν:R→R can be described as the max-min value of a two-stage game parameterised by a real number *h*. In the first stage, Player 1 (the maximiser) chooses a mixed action $p\in \Delta (A_{1})$ under the restriction that H(p)≥h or H(p)=0. In the second stage, Player 2 (the minimiser) responds with an action $a^{2}\in A_{2}$. The max-min value of that game is ν(h). Formally, the function ν is defined by

$$\nu \left(h\right)=\max_{\begin{array}{c} p\in \Delta (A^{1}):\\ H(p)\geq h\;\mathrm{or}\\ H(p)=0 \end{array}}\;\min_{a^{2}\in A_{2}}{\mathrm{error}}_{p(a^{1})}u\left(a^{1},a^{2}\right).$$

Recall that we denote the mixed (min-max or max-min) value of a two-person zero-sum game *G* by $Val^{\prime }G$. We have the following asymptotic estimate.

**Theorem** **3** ([9])**.** *For every ε>0, there exists $k_{0}\in N$ such that for any $k_{0}\leq k^{1}\leq k^{2}\leq T$,*

$$\nu \left(\frac{\log k^{2}}{k^{1}}+\varepsilon \right)-\varepsilon \leq Val^{\prime }G^{T}\left[k_{obl}^{1},k^{2}\right]\leq Val^{\prime }G^{T}\left[k^{1},k^{2}\right]\leq \nu \left(\frac{\log k^{2}}{k^{1}}-\varepsilon \right)+\varepsilon .$$

The proof of Theorem 3 relies heavily on a characterisation of the entropy of Bernoulli shifts that might be of independent interest. The entropy is characterised in terms of the length of a cycle that can be implemented through oblivious bounded recall strategies. The set of periodic sequences that can be implemented thorough oblivious *k*-recall strategies is denoted

$$A\left[k\right]=\left\{\bar{a}\in A^{Z}:\;\left(a_{t},\ldots ,a_{t+k-1}\right)=\left(a_{s},\ldots ,a_{s+k-1}\right)\Rightarrow a_{t+k}=a_{s+k}\;\forall s,t\in Z\right\}.$$

The set of *n*-periodic (two sided) infinite sequences over *A* is denoted $A^{\left(n\right)}\subset A^{Z}$. For $\bar{a}\in A^{N}$, the element of $A^{\left(n\right)}$ that identifies with $\bar{a}$ on the first *n* elements is denoted ${\bar{a}}^{\left(n\right)}=\ldots ,a_{1},a_{2},\ldots ,a_{n},a_{1},a_{2},\ldots ,a_{n},\ldots$.

**Theorem** **4.** *Let $\bar{X}=X_{1},X_{2},\ldots$ be a sequence of i.i.d. random variables that take values in a finite set A.*

$$\sup\left\{h:\lim_{k\to \infty }\frac{\log P\left({\bar{X}}^{\left(\lfloor \exp(hk)\rfloor \right)}\in A\left[k\right]\right)}{\exp(hk)}=0\right\}=H\left(X_{i}\right).$$

**Problem** **2.** *We wonder whether and how Theorem 4 extends to other ergodic processes.*

The easier part of Theorem 4 is showing that the supremum is not greater than the entropy. To this end, one can utilise two claims. The first claim is that the empirical frequency of ${\bar{X}}^{\left(n\right)}$ converges in probability to the distribution of $X_{i}$ exponentially fast. The second is the following claim.

**Claim** **1.** *For any k, n and $\bar{a}\in A\left[k\right]\cap A^{\left(n\right)}$, $n\leq \exp(H\left(\mathrm{emp}\left(\bar{a}\right)\right))$.*

**Proof.**  Let *t* be a random variable that distributes uniformly in {1,…,n}. Let $Y_{1},\ldots ,Y_{n}$ be random variables defined by $Y_{i}=a_{t+i}$. Consider the random variable $Y=(Y_{1}\ldots ,Y_{k})$. Since $\bar{a}\in A\left[k\right]\cap A^{\left(n\right)}$, *Y* is uniformly distributed in a set of size *n*; therefore H(Y)=log(n). The distribution of each $Y_{i}$ is $\mathrm{emp}(\bar{a})$; therefore $H\left(Y\right)\leq H\left(Y_{1}\right)+\cdots +H\left(Y_{k}\right)=H\left(\mathrm{emp}\left(\bar{a}\right)\right)k$. □

**Remark** **5.** *The history behind Theorem 3 is as follows. Lehrer [10] proved two special cases: the case of $\frac{\log k^{i}}{k^{j}}$ converging to 0, namely, if $\lim_{n\to \infty }\frac{\log k^{1}\left(n\right)}{k^{2}\left(n\right)}=\lim_{n\to \infty }\frac{\log k^{2}\left(n\right)}{k^{1}\left(n\right)}=0$, then $\lim_{n\to \infty }Val^{\prime }G^{\infty }\left[k^{1}\left(n\right),k^{2}\left(n\right)\right]=\lim_{n\to \infty }Val^{\prime }G^{\infty }\left[k_{obl}^{1}\left(n\right),k^{2}\left(n\right)\right]=Val^{\prime }G$; and the case of $\frac{\log k^{2}}{k^{1}}$ being sufficiently large, namely, there exists a number L>0 (that depends on G) such that if $\lim_{n\to \infty }\frac{\log k^{2}\left(n\right)}{k^{1}\left(n\right)}>L$, then $lim_{n\to \infty }Val^{\prime }G^{\infty }\left[k^{1}\left(n\right),k^{2}\left(n\right)\right]=ValG$.*

*Neyman and Okada [2] extended Lehrer’s result by finding two numbers 0<O<N<L (depending on G), such that (asymptotically) if $\frac{\log k^{2}}{k^{1}}<O$, then $Val^{\prime }G^{\infty }\left[k_{obl}^{1},k^{2}\right]\geq Val^{\prime }G-o\left(1\right)$ and if $\frac{\log k^{2}}{k^{1}}>N$, then $Val^{\prime }G^{\infty }\left[k^{1},k^{2}\right]=ValG+o\left(1\right)$.*

### More than Two Players

From two-person games one may proceed to study three-person games. The quantity that we consider is the mixed min-max value, a.k.a. the individually rational level. This quantity is important since it is an ingredient in the construction of equilibrium strategies.

For the sake of exposition, we define a three-person game $G=\langle A=A_{1}\times A_{2}\times A_{3},u:A\to R\rangle$ similarly to our definition of a two-person zero-sum game. We think of *u* as the payoff of Player 1. We disregard the payoffs of the other players (since we consider the min-max value of player 1 only). The mixed min-max value (of Player 1) is defined by

$$Val^{\prime }G=\min_{\left(p,q\right)\in \Delta \left(A_{2}\right)\times \Delta \left(A_{3}\right)}\max_{a^{1}\in A_{1}}{\mathrm{error}}_{p\left(a^{2}\right)\times q\left(a^{3}\right)}u\left(a_{1},a_{2},a_{3}\right).$$

The correlated min-max value (of Player 1) is defined by

$$Val^{\prime \prime }G=\min_{p\in \Delta (A_{2}\times A_{3})}\max_{a^{1}\in A_{1}}{\mathrm{error}}_{p(a^{2},a^{3})}u\left(a_{1},a_{2},a_{3}\right).$$

The game $G^{T}\left[k^{1},k^{2},k^{3}\right]$ is defined as the *T*-fold repetition of *G* where each player *i* is restricted to $k^{i}$-recall strategies. Our holy grail is the question

$$Val^{\prime }G^{T}\left[k^{1},k^{2},k^{3}\right]=?$$

The answer is not known even in the symmetric case where all the players have the same recall capacity. To pinpoint the problem, one may focus on a very specific game.

**Problem** **3.** *Consider the three-person Matching Pennies Game $G=\langle A=\left\{0,1\right\}\times \left\{0,1\right\}\times \left\{0,1\right\},1-1_{\left\{a^{1}=a^{2}=a^{3}\right\}}\rangle$.*

$$\lim_{k\to \infty }Val^{\prime }G^{\infty }\left[k,k,k\right]=?$$

*and does the limit exist?*

What is known about this problem? From the analysis of two-person games (e.g., Reference [10] or Theorem 3) one can infer that for every three-person game *G* and T≥k,

$$Val^{\prime \prime }G-o\left(1\right)\leq Val^{\prime }G^{T}\left[k,k,k\right]\leq Val^{\prime }G+o\left(1\right).$$

The first inequality follows from strengthening Players 2 and 3 by allowing them to cooperate as if they were one player. The second inequality follows from weakening 2 and 3 by restricting them oblivious strategies.

But where does the truth lie? Is it closer to min-max value of the one stage game, or is it closer to the correlated min-max value?

**Remark** **6.** *Historically, following Reference [10], researchers were tempted to believe that it is closer to the uncorrelated value. The researchers’ belief was based on the intuitive assumption that since Player 1 can observe the states of 2 and 3, they should not be able to share any secret between them without Player 1 noticing and, therefore, they could not correlate their strategies against Player 1.*

*Bavly and Neyman [11] were the first to challenge the above intuition. They introduced a fourth player, who is much stronger than the other players but whose actions are irrelevant for the payoff. In their modified model, they showed that the min-max value of the repeated game converges to the correlated min-max value of the one-stage game. Their work motivated the search for a counterexample, a game for which the min-max value of the repeated game is close to the correlated min-max value of the one-stage game.*

The following theorem shows that in some games the min-max value of the repeated game is close to the correlated min-max value of the one-stage game.

**Theorem** **5.** *([12]) For every three-person game G and every C,ε>0, by cloning any one of the actions of either Player 2 or 3 sufficiently many times, we obtain a game $\hat{G}$ in which*

$$Val^{\prime }{\hat{G}}^{\infty }\left[Ck,k,k\right]\leq Val^{\prime \prime }\hat{G}+\varepsilon ,$$

*for any k sufficiently large.*

A following converse theorem says that for any fixed game *G*, there exists *C* sufficiently large so that the min-max value of the repeated game is arbitrarily close to the min-max value of the one-stage game.

**Theorem** **6** ([13])**.** *For every three-person game G and every C,ε>0,*

$$Val^{\prime }G^{T}\left[Ck,k,k\right]\geq Val^{\prime }G-2\sqrt{\log(|A|)/C}-\varepsilon ,$$

*for any k sufficiently large and any T≥k.*

Theorem 5 relies on a characterisation of the distributions of actions that Players 2 and 3 can implement without looking at the actions of Player 1. Note that, since not allowing Players 2 and 3 to look at Player 1’s actions weakens Players 2 and 3, this characterisation implies an upper bound on the min-max value. In order to describe the characterisation we consider a game with just two players (who represent Players 2 and 3 in the original game).

**Definition** **1.** *Let $A_{1}$, $A_{2}$ be finite alphabets, $p\in \Delta (A_{1}\times A_{2})$ and $k^{1},k^{2},m:N\to N$. We use the notations of repeated games with bounded recall as if we referred to a two-person game $G=\langle A_{1}\times A_{2},u\rangle$ for some payoff function u. We say that p is *implementable* for a duration m through recall capacities $k^{1}$ and $k^{2}$ if there exist $\sigma_{n}^{i}\in \Delta (\Sigma^{i}\left[k^{i}\left(n\right)\right]$ (i=1,2, n∈N) such that the play induced by $\sigma_{n}^{1}$ and $\sigma_{n}^{2}$, $a_{1},a_{2},\ldots$, satisfies*

- $∥\mathrm{emp}(a_{t+1},\ldots ,a_{t+m})-p∥=o\left(1\right)$, and
- $\frac{1}{m}H\left(a_{t+1},\ldots ,a_{t+m}\right)\geq H\left(p\right)-o\left(1\right)$,

*for any t sufficiently large.*

The following theorem characterises the implementable distributions.

**Theorem** **7** ([12])**.** *Given the notation of Definition 1, denote by $p_{i}$ the marginal of p on $A_{i}$ (i=1,2), and the mutual information that p induces I(p). Suppose $\lim_{n\to \infty }m\left(n\right)=\infty$, and suppose the triple $\left(k^{1}\left(n\right),k^{2}\left(n\right),m\left(n\right)\right)$ is asymptotically proportional to some $\left(\kappa^{1},\kappa^{2},1\right)$, then a sufficient condition for implementability is*

$$\kappa^{1}H\left(p_{1}\right)+\kappa^{2}H\left(p_{2}\right)\geq I\left(p\right).$$

*This condition is also necessary, if $\kappa^{1}=\kappa^{2}$.*

## 4. Entropy in Economics

### 4.1. Value of Information

#### 4.1.1. Experiments

Making decisions when the only information-gathering tools available are noisy signal sets is an unavoidable aspect of life. Examples are not difficult to adduce. In a bank run situation, one may be inclined to withdraw one’s funds from a bank if there are rumours that the fundamentals of the bank are weakening, but how is one to ascertain whether the rumours are true? One could rely on several alternative information sources, such as official bank spokesperson statements, media reports, the length of queues at the bank, and additional rumours whispered by neighbours. Each source is ‘noisy’ in the sense of giving certain signals with positive probability no matter what is the true state of the bank, and it may be unclear which source is to be preferred.

**Figure 1:**
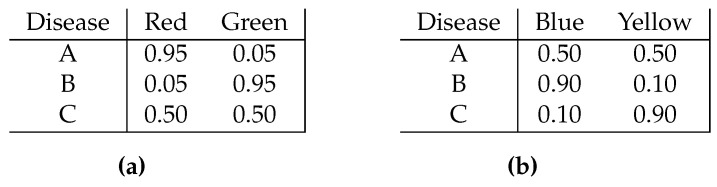
Laboratory tests with false positive and negatives.

Another example is given by laboratory tests for the diagnosis of an illness; such tests may not be entirely one hundred percent reliable if they indicate false positives or false negatives. Imagine a patient exhibiting symptoms that are consistent with three possible diseases, stumping a medical staff worthy of television’s Dr House. Two medical laboratory tests are available, which turn a chemical into one of two colours, depending on which disease is the true one. These are exhibited in Figure 1; for example, in test (a), if the true disease is disease B then the chemical will turn red with probability 0.05 and green with probability 0.95. Unfortunately, no matter what is the true state, and under both tests, no matter which colour appears there will still remain some uncertainty as to which of A, B, or C is the true case. Given the choice of conducting only one of these tests, which should the medical staff choose? Which one is more valuable from the perspective of the information it supplies?

To begin answering such questions rigorously, one needs to decide on a criterion for measuring the value of information provided by ‘noisy signals’. Suppose that Ω is a (finite) set of possible states. An *experiment* (also called an information structure, or signal structure in the literature) over Ω is a pair (ψ,S) where *S* is a finite set of signals and $\psi ={\left\{\psi_{\omega ,s}\right\}}_{\omega \in \Omega ,\;s\in S}$ is a stochastic matrix, with the rows representing the states and the columns the signals.

It is assumed that every signal *s* has positive probability under at least one state. This is without loss of generality; if it does not hold a signal that has zero probability of being received can simply be removed from the set of signals. When the true state is ω the decision maker receives signal *s* with probability $\psi_{\omega ,s}$. The two matrices depicted in Figure 1 satisfy the definition, with the state space being {A,B,C} in both cases and pairs of colours serving as the signal spaces.

A decision maker with a prior distribution π∈Δ(Ω) over the the states can use an experiment ψ to arrive at a posterior distribution following receipt of a signal in *S*. In detail, given prior π, the total probability of seeing s∈S under ψ is $p_{\psi }^{\pi }\left(s\right)=\sum_{\omega \in \Omega }\pi \left(\omega \right)\psi_{\omega ,s}$. Then the posterior probability distribution $q_{\psi }^{\pi }\left(\cdot |s\right)\in \Delta \left(\Omega \right)$ conditional on signal *s* is calculated by Bayes’ Rule such that for each state ω∈Ω:

(2) $$q_{\psi }^{\pi }\left(\omega |s\right)=\frac{\pi \left(\omega \right)\psi_{\omega ,s}}{p_{\psi }^{\pi }\left(s\right)}.$$

Denote the collection of all experiments over Ω by I(Ω). An ordering ${≻}_{O}$ of I(Ω) will be termed a *value of information* ordering. The intention is that if $\psi_{1}{≻}_{O}\psi_{2}$ then $\psi_{1}$ has greater value as a source of information for discovering the true state than $\psi_{2}$. Many orderings are possible; the challenge is to identify an ordering that captures our intuition regarding when one experiment truly provides more informational value than another.

#### 4.1.2. The Blackwell Ordering of Experiments

David Blackwell ([14,15]), in seminal work that has had a significant impact on the literature, suggested a (partial) ordering of experiments using a decision theoretic criterion. Let (A,u) be a decision making problem over the state space Ω; that is, the utility function is given as u:Ω×A→R. We assume that all decision makers are utility maximisers. Hence if a decision maker knows that the state of the world is ω∈Ω the action chosen will be ${\mathrm{argmax}}_{a\in A}u\left(\omega ,a\right)$.

If the decision maker cannot observe the state directly but has access to an experiment ψ that supplies a signal *s* conditional on the state, the best that the decision maker can do is implement a policy, by which we mean a mixed strategy conditional on the signal, that is, β(s)∈Δ(A). Denote the collection of all possible policies by B:={β∣β:S→Δ(A)}.

The situation as described now is that there is a mapping ψ:Ω→S and a mapping β:S→Δ(A). Composition of these mappings yields β∘ψ:Ω→Δ(A), where β∘ψ(·|ω) denotes the probability of choosing an action conditional on the true state being ω.

A decision maker with a prior belief π∈Δ(Ω) faced with a decision making problem (A,u) can calculate the expected payoff of a policy β, contingent on information structure ψ, by way of

$$E\left(\beta ;\pi ,A,u,\psi \right)=\sum_{\omega \in \Omega }\pi \left(\omega \right)\left(\sum_{a\in A}u\left(a,\omega \right)\beta \circ \psi \left(a|\omega \right)\right),$$

and then select an optimal policy $\hat{\beta }$. The payoff of ψ for decision maker (A,u) with prior π using $\hat{\beta }$ is then given by $\max_{\beta \in B}E\left(\beta ;\pi ,A,u,\psi \right).$

Suppose that we wish to remove the dependence on the prior in the above reasoning. For a pair $\psi_{1},\psi_{2}\in I\left(\Omega \right)$ declare that $\psi_{1}$ is *subjectively more informational* for decision problem (A,u) than $\psi_{2}$ if

$$\max_{\beta }E\left(\beta ;\pi ,A,u,\psi_{1}\right)\geq \max_{\beta }E\left(\beta ;\pi ,A,u,\psi_{2}\right)$$

for *all* priors π∈Δ(Ω).

Next, to get an interpersonally objective ordering, define $\psi_{1}$ to be *more Blackwell informative* than $\psi_{2}$, denoted $\psi_{1}{⪰}_{B}\psi_{2}$, if $\psi_{1}$ is subjectively more informational than $\psi_{2}$ for *every* possible decision problem (A,u). This value-of-information ordering ${⪰}_{B}$ is the Blackwell ordering.

The nice aspect of the Blackwell ordering is that it is ‘objective’ in the sense that if $\psi_{1}{≻}_{B}\psi_{2}$ then every rational decision maker will agree that $\psi_{1}$ has greater informational value than $\psi_{2}$ no matter what decision making problem is at issue and no matter what prior distribution is assumed. The drawback to this very strong requirement of ‘objective unanimity’ amongst decision makers is that we pay the heavy price of working with a very partial ordering (in fact it is only a pre-order). In other words, not all experiments can be ordered by the Blackwell ordering; given two experiments $\psi_{1}$ and $\psi_{2}$ one may discover that they are incomparable under this ordering. In that case the Blackwell ordering is silent regarding the question which experiment provides greater value of information.

To see this, let Ω={A,B,C}, $S=\{s_{1},s_{2}\}$, and let $\psi_{1}$ be the matrix of Figure 1a, where $s_{1}=Red$ and $s_{2}=Green$, while $\psi_{2}$ is the matrix of Figure 1b, where $s_{1}=Blue$ and $s_{2}=Yellow$. Alongside these, define action set $A=\{\alpha_{1},\alpha_{2}\}$ and payoff functions $u_{1}$ and $u_{2}$ such that $u_{1}\left(A,\alpha_{1}\right)=u_{1}\left(B,\alpha_{2}\right)=u_{2}\left(B,\alpha_{1}\right)=u_{2}\left(C,\alpha_{2}\right)=1$, with payoff zero for all other combinations of states and actions for both $u_{1}$ and $u_{2}$. Let $p_{1}=\left(0.5-\varepsilon ,0.5-\varepsilon ,2\varepsilon \right)$ and $p_{2}=\left(2\varepsilon ,0.5-\varepsilon ,0.5-\varepsilon \right)$ be priors over Ω.

Then it is fairly straight-forward to see that, for sufficiently small ε, a decision maker facing the decision making problem characterised by payoff $u_{1}$ and prior $p_{1}$ will prefer $\psi_{1}$ to $\psi_{2}$. This is because in this situation under both $\psi_{1}$ and $\psi_{2}$, receiving signal $s_{1}$ instructs optimally choosing action $a_{1}$ and signal $s_{2}$ instructs optimally choosing $a_{2}$. Taken together, the optimal expected payoff from $\psi_{1}$ is higher than that from $\psi_{2}$. By similar reasoning, exactly the reverse holds for payoff $u_{2}$ and prior $p_{2}$: here, the optimal expected payoff from $\psi_{2}$ is higher than that from $\psi_{1}$. Hence $\psi_{1}$ and $\psi_{2}$ are incomparable in the Blackwell ordering.

#### 4.1.3. Entropy and the Value of Information for Investors

The partiality of the Blackwell ordering has motivated several researchers to seek an extension of the Blackwell ordering to a total ordering of all experiments. One suggested completion, due to Cabrales, Gossner, and Serrano [16], relates experiments to entropy, investments, and measures of risk.

Let π∈Δ(Ω). Since π is a distribution, one can calculate its entropy $H\left(\pi \right)=\sum_{\omega \in \Omega }\pi \left(\omega \right)\ln\left(\pi \left(\omega \right)\right)$. Next, recall that by Equation (2) each signal *s* defines a posterior distribution $q_{\psi }^{\pi }\left(\cdot |s\right)\in \Delta \left(\Omega \right)$. Each such posterior distribution, in turn, has its own entropy $H(q_{\psi }^{\pi }\left(\cdot |s\right))$. One can then define from this the weighted mean posterior entropy $\sum_{s\in S}p_{\psi }^{\pi }\left(s\right)H\left(q_{\psi }^{\pi }\left(\cdot |s\right)\right)$, where, as before, $p_{\psi }^{\pi }\left(s\right)$ is the total probability of signal *s*, given the prior π.

Using these concepts, Reference [16] defines what they term the *entropy informativeness* of ψ (relative to prior π) to be the difference between the entropy of the prior and the weighted mean posterior entropy:

(3) $$I\left(\psi ,\pi \right):=H\left(\pi \right)-\sum_{s\in S}p_{\psi }^{\pi }\left(s\right)H\left(q_{\psi }^{\pi }\left(\cdot |s\right)\right).$$

If we attach to entropy the interpretation that it measures uncertainty, a reduction in entropy corresponds to reduction in uncertainty. Hence I(ψ,π) may be considered a measure of the increase in information supplied by the experiment ψ relative to the base-line information in the prior π, as given by the reduction in uncertainty quantified in the difference in entropy measurement expressed in Equation (3).

It can readily be shown that I(ψ,π) is always a positive real number. Since the real numbers have a natural ordering, and for fixed π we may regard I(·,π) as mapping each ψ∈I to a real number, it follows immediately that I(·,π) defines a total ordering of experiments. To see further that the entropy informativeness ordering extends the Blackwell ordering, consider the following economic interpretation of I(ψ,π) put forth in Reference [16].

Let the set of states of the world Ω be identified with a set of integers K={1,…,k}. From here on, fix a prior π∈Δ(K). In the model, each decision maker is identified with a concave and twice continuously differentiable utility function *u* for money. Every decision maker is also endowed with an initial wealth level *w*. Each pairing of *u* and *w* determines an *Arrow-Pratt coefficient of relative risk aversion*, defined as

$$\rho_{u}\left(w\right)=-w\frac{u^{\prime \prime }\left(w\right)}{u^{\prime }\left(w\right)}.$$

It will further be assumed that all decision makers satisfy the property of having *increasing relative risk aversion (IRRA)*, which in this context can be taken to mean that ρ is non-decreasing as a function of *w*. In Reference [16] it is also assumed that all decision makers are *ruin averse*, meaning that it always holds that $\lim_{w\to 0^{+}}u\left(w\right)\to -\infty$. The collection of utility functions satisfying all of these properties is denoted $U^{*}$.

An *asset* is defined to be an element $b\in R^{\left|K\right|}$, interpreted as meaning that at each realised state *k* the asset *b* pays $b_{k}\in R$. An asset is a *no-arbitrage* asset if $\sum_{k}\pi \left(k\right)b_{k}\leq 0$. Denote the set of no-arbitrage assets by $B^{*}$; this is the set of *investment opportunities* presented to the decision makers.

When a decision maker with initial wealth *w* chooses investment $b\in B^{*}$ and state *k* is realised, the updated wealth becomes $w+b_{k}$. Since $B^{*}$ includes $0_{K}$, the vector in $R^{\left|K\right|}$ consisting of zeros in every position, decision makers always have the option of inaction, guaranteeing that they can maintain wealth *w*. We do, however, impose the constraint that bankruptcy (the possibility of negative wealth) is not allowed.An investment *b* is termed *feasible* at wealth *w* when $w+b_{k}\geq 0$ in every state *k*. Denote by $B_{w}^{F}$ the set of investment opportunities that are feasible at wealth *w*.

Just before deciding which investment opportunity to pick, each decision maker is offered the opportunity to purchase experiment ψ at price μ. The question posed here is what price the decision maker would be willing to pay for the information supplied by ψ. Note first that the expected utility of *b* for a decision maker with utility *u*, initial wealth *w*, and prior belief π (without the benefit of the information supplied by any experiment) is

(4) $$V\left(u,w,\pi \right):=\underset{b\in B_{w}^{F}}{\sup}\sum_{k}\pi \left(k\right)u\left(w+b_{k}\right).$$

Since we have supposed that decision makers are risk averse and that bankruptcy is forbidden, the optimal choice of Equation (4), absent any additional information, is inaction. Hence,

V(u,w,π)=u(w).

If ψ is made available, then the expected payoff increases to

$$\gamma \left(\psi ,u,w,\pi \right):=\sum_{s\in S}p_{\psi }^{\pi }\left(s\right)V\left(u,w,q_{\psi }^{\pi }\left(\cdot |s\right)\right),$$

where $p_{\psi }^{\pi }\left(s\right)$ is the probability of seeing *s* and $q_{\psi }^{\pi }$ is the posterior probability distribution over the states upon receipt of *s*; in other words, γ(ψ,u,w,π) is the weighted average of the expected utilities of the posterior distributions, with weighting given by the probabilities of seeing the signals. It follows that the gain from making use of ψ is

γ(ψ,u,w,π)−u(w),

and that if ψ is offered to be purchased at price μ the the decision maker will *accept* the offer if

γ(ψ,u,w−μ,π)>u(w),

and *reject* it otherwise.

Define $\psi_{1}$*investment dominates*$\psi_{2}$ if for every wealth *w* and price μ<w, if $\psi_{1}$ is rejected by all decision makers with utility $u\in U^{*}$ then $\psi_{2}$ is also rejected by all those same decision makers.

**Theorem** **8** ([16]). *For each prior π, experiment $\psi_{1}$ investment dominates experiment $\psi_{2}$ if and only if $I\left(\psi_{1},\pi \right)\geq I\left(\psi_{2},\pi \right)$.*

A sketch of the proof idea for Theorem 8 is as follows. As a first step, Reference [16] shows that the utility function ln(w) for all wealth levels *w* is representative for investment dominance in the sense that $\psi_{1}$ investment dominates $\psi_{2}$ if and only if for every *w* and μ<w a decision maker with the ln utility function who rejects $\psi_{1}$ also rejects $\psi_{2}$. They then show that a decision maker with logarithmic utility will determine that $\psi_{1}$ investment dominates $\psi_{2}$ precisely when the entropy informativeness of $\psi_{1}$ dominates that of $\psi_{2}$, as defined in Equation (3).

It is a corollary of Theorem 8 that if $\psi_{1}$ is more Blackwell informative than $\psi_{2}$ then $I\left(\psi_{1},\pi \right)\geq I\left(\psi_{2},\pi \right)$ for all π. This is because $\psi_{1}{≻}_{B}\psi_{2}$ implies that no matter what the utility function, the prior, or the decision making problem, a decision maker using $\psi_{1}$ can obtain greater expected payoff than with $\psi_{2}$; hence $\psi_{1}$ certainly investment dominates $\psi_{2}$.

Putting it all together, the Cabrales–Gossner–Serrano entropy ordering is a value of information ordering of experiments that extends the Blackwell informativeness ordering to a total ordering. There is, however, one drawback to the entropy ordering. By definition, I(ψ,π) measures the reduction in entropy from the entropy of the prior π to the expected entropy of the generated posteriors. If a different prior $\pi^{\prime }$ is used, the entropy reduction measured by $I(\psi ,\pi^{\prime })$ may be very different. Does this mean that the entropy ordering is unavoidably sensitive to the prior being used for this measurement?

Reference [16] answers this question in the affirmative: there are examples in which the choice of a different prior leads to different orderings of I (although of course all of those different orderings extend the Blackwell ordering). Even worse, the paper proves that there exists no index that orders information structures that is both compatible with investment dominance and independent of the agent’s prior. (Cf. [17]) In other words, almost every one of the infinite number of possible priors defines a different ordering.

### 4.2. Rational Inattention

In the most ideal presentation of the classical theory of economies composed of perfectly rational agents, each of whom is a *homo economicus*, all relevantly available economic information is immediately known and analysed by all agents, who instantaneously select optimal actions in response to attain economic equilibria. The implications of such a theory are many; amongst them are that business cycles are an impossibility, since prices and wages react instantaneously and optimally to changes in economic conditions and supply and demand. As a result, employment and output always remain at optimal levels with respect to objective economic fundamentals.

This view is challenged by Keynesian economic theories. One of the features of such theories is the assertion of ’price stickiness’, which can be presented as an assertion that prices and wages react with considerable delay to changes in underlying economic fundamentals. Since delays cause prices and wages to be suboptimal, economies are slow to attain market clearing equilibria and hence undergo periods of underperformance in contrast to what would be expected by a seamless model of continuously optimising agents.

An oft-repeated critique of the price stickiness assumption in Keynesian models is that it is inserted into formal economic models *ad hoc*, without micro-foundational justification. To contend with this, several explanations have been proffered in the literature. These include assumptions that agents may experience signal-extraction difficulties in distinguishing movements in aggregate prices and wage movements in the specific prices they encounter in transactions. Other theories seek explanations in models of behavioural deviations from full rationality and in bounded rationality models.

We focus here on the *rational inattention* model, initiated in a seminal paper due to Christopher Sims ([18]. (Nearly all of the material presented in this section is taken either from Reference [18] or from Reference [19].) This model begins with the observation that people react sporadically and imperfectly to the information they receive. Even individuals who actively read charts on financial data often fail to take actions based on all the information they have seen, and typically react only when data strike them as saliently unusual. In contrast, one would expect fully rational economic agents to be making fine adjustments to their spending schedules, investment portfolios, and other economic activities on a continual basis given any received information on changes in market parameters, no matter the amplitude of the changes.

The explanation given to this by the rational inattention model is that the benefits of continuous adjustment are slight relative to the extent of attention required to implement them; people have more important things to think about day to day and moment to moment than the tiny gains they might attain from continuous monitoring of economic variables. Rational inattention presumes that the ability of individuals to translate external data into actions is constrained by a finite Shannon channel capacity inherent to humans for information processing.

To begin modelling this mathematically, recall the definition of I(X,Y), the mutual information(Not to be confused with the similar notation of Equation (3), which refers to entropy informativeness in a different context.) between two probability distribution functions *X* and *Y*. This is the difference between the expected value of the log of the joint probability distribution of *X* and *Y* and the sum of the two expected values of the logs of the marginal probability distributions of *X* and *Y*. The mutual information measure is crucial for the development of the concepts related to Shannon channel capacity.

A channel, in Shannon’s theory, is a description of possible inputs (in terms of probability distributions) and of conditional distributions of inputs given outputs. The exact distribution of the inputs, however, may be chosen judiciously. More to the point, if one chooses the distribution of the inputs to maximise the mutual information between input distribution and output distribution, the channel is transmitting information at maximal capacity.

The coding theorem states that inputs can be coded in such a way that information can always be transmitted through the channel with arbitrarily low error rates and arbitrarily close the the channel capacity transmission rate. This does, however, come with a price: coding almost always introduces delay.

Suppose our objective is to minimise $E[{\left(Y-X\right)}^{2}]$, where *Y* is the action to be chosen by the economic agent and *X* is a random variable can only be observed through a finite-capacity channel, with capacity κ. What is the optimal choice for the conditional distribution of Y∣X subject to the requirement that the information flow about *X* required to generate *Y* is finite? Formally, supposing that p(·) is given as the probability density function of *X* and that we seek the optimal q(·) associated with *Y*, the mathematical task is

$$\begin{array}{cc} \min_{q}\{ & E\left[{\left(Y-X\right)}^{2}\right]=\int {\left(y-x\right)}^{2}q\left(y|x\right)p\left(x\right)\;dy\;dx\}\\ \; & s.t.\int q(y|x)\;dx=1\;\mathrm{for}\;\mathrm{all}\;x\\ \; & \;\mathrm{and}\;-E\left[E\left[\log_{2}q\left(Y|X\right)|X\right]\right]+E\left[\log_{2}\int q\left(Y|x\right)p\left(x\right)dx\right]<\kappa \end{array}$$

Reference [18] then shows that if *X* is distributed X∼N then the optimal form of *q* is Gaussian and *X* and *Y* are jointly normal. In that case, this is equivalent to observing *X* with an error.

To take one economic example to which this can relate, consider a permanent income calculation. The permanent income hypothesis, first proposed by Milton Friedman, postulates that individuals determine their consumption at each point in time *t* not only in relation to present wealth at time *t* but also taking into consideration expected income in future time periods. This has strong implications for savings and consumption rates. In particular, it predicts consumption smoothing, spreading out of spending over time.

In a simple formal model of permanent income calculation, postulate an infinitely-lived agent who maximises his or her life-time utility from the consumption of a stream of consumption (with consumption at time *t* denoted by $C_{t}$). The utility function of consumption is presumed to be $u\left(C_{t}\right)=C_{t}-\frac{1}{2}C_{t}^{2}$.

At the start of each period *t*, the agent has $W_{t}$ available from which to consume $C_{t}$, leaving $W_{t}-C_{t}$, which then grows each period in accordance with a gross interest rate *R*. We may also suppose that the agent has labour or endowment income available at time *t*, which is denoted $Y_{t}$. The utility of consumption in future periods is discounted at a fixed rate β per time period.

The objective of the agent is

$$\begin{array}{cc} \max_{{\left\{C_{t}\right\}}_{t=0}^{\infty }} & E\left[\sum_{t=0}^{\infty }\beta^{t}u\left(C_{t}\right)\right]\\ =\max_{{\left\{C_{t}\right\}}_{t=0}^{\infty }} & E\left[\sum_{t=0}^{\infty }\beta^{t}\left(C_{t}-\frac{1}{2}C_{t}^{2}\right)\right]\\ \; & \;s.t.\;W_{t+1}=R\left(W_{t}-C_{t}\right)+Y_{t+1}. \end{array}$$

So far this is a standard permanent income calculation. The text-book solution to the model as presented up to here is

(5) $$C_{t}=\beta W_{t}+\left(1-\beta \right)\bar{Y}.$$

Suppose now that distributions are Gaussian, $W_{0}∼N\left(\bar{W},\sigma_{W}^{2}\right)$, and $Y_{t}∼N\left(\bar{Y},\sigma_{Y}^{2}\right)$ i.i.d. for all *t*, where $\bar{W}$ and $\bar{Y}$ stand for mean values. And to relate it all to rational inattention, suppose that $I_{t}$ denotes the information available at time *t*, with all variables known at time *t* measurable with respect to $I_{t}$. Consistent with previous model assumptions, we suppose that updating from $W_{t}|I_{t-1}$ to $W_{t}|I_{t}$ occurs with upper bound channel capacity κ.

Sims [18] shows that under these assumptions the agent will behave as if observing noisy state measurements; more precisely the consumption sequence $\left\{C_{t}\right\}$ will satisfy

(6) $$C_{t}=\beta W_{t}^{*}+\left(1-\beta \right)\bar{Y},$$

where $W_{t}^{*}=W_{t}+\zeta_{t}$ such that $\zeta_{t}$ is an i.i.d. normal noise factor.

Comparing Equations (5) and (6), the latter adds the noise term $\zeta_{t}$ that is not present in Equation (5) and is not inherent to the original variables of the model. The extra noise can have significant cumulative effects on consumption and savings schedules.

Rational inattention models have been studied in much greater generality than those involving quadratic objective functions and Gaussian distributions (see References [20,21,22,23]). This literature is too broad to be fully surveyed here; we will provide only a very brief summary of one of its main implications.

The solutions to the models in the above papers often imply a discrete distribution for agent actions, even when the external uncertainty is continuously distributed. In the context of product prices, which is most interesting because of its implications for disputes regarding the ‘stickiness’ of prices, the results point to prices that jump amongst a finite set of values when they change, which is consistent with data observations but is not fully explained by models other than rational inattention models.

If price setters who are rationally inattentive adapt to available information only occasionally and select only from a finite set of possible prices, then price setters are far from the image of agents who continuously and optimally react to every revelation of economic information. In particular, their response to monetary policy changes will differ from that postulated in classical rational expectations models.

## 5. More on Entropy in Repeated Games

The previous sections depicted a small (and rather biased towards the interest of the authors) selection of the uses of entropy in game and economic theory. We have not mentioned a few important research trends to which we would like to provide references for further reading. Repeated zero-some games with a limited source randomness were studied by Neyman and Okada [1,24] and later by Gossner and Vieille [25]. Correlation based on an exogenous source of randomness was studied in Reference [26]. Valizadeh and Gohari [27] showed how to overcome information leakage in the above mentioned settings. Another fruitful research direction is the communication game introduced by Gossner, Hernández, and Neyman [28] which was followed up by, for example, Cuff and Zhao[29], Larrousse, Lasaulce, and Wigger [30], and Le Treust [31].
